# The Influence of Action Video Gaming Experience on the Perception of Emotional Faces and Emotional Word Meaning

**DOI:** 10.1155/2021/8841156

**Published:** 2021-05-22

**Authors:** Yuening Yan, Yi Li, Xinyu Lou, Senqi Li, Yutong Yao, Diankun Gong, Weiyi Ma, Guojian Yan

**Affiliations:** ^1^The Clinical Hospital of Chengdu Brain Science Institute, MOE Key Lab for Neuroinformation, University of Electronic Science and Technology of China, Chengdu, China; ^2^Center for Information in Medicine, School of Life Science and Technology, University of Electronic Science and Technology of China, Chengdu, China; ^3^Faculty of Natural Science, University of Stirling, Stirling, UK; ^4^School of Human Environmental Sciences, University of Arkansas, Fayetteville, AR, USA

## Abstract

Action video gaming (AVG) experience has been found related to sensorimotor and attentional development. However, the influence of AVG experience on the development of emotional perception skills is still unclear. Using behavioral and ERP measures, this study examined the relationship between AVG experience and the ability to decode emotional faces and emotional word meanings. AVG experts and amateurs completed an emotional word-face Stroop task prior to (the pregaming phase) and after (the postgaming phase) a 1 h AVG session. Within-group comparisons showed that after the 1 h AVG session, a more negative N400 was observed in both groups of participants, and a more negative N170 was observed in the experts. Between-group comparisons showed that the experts had a greater change of N170 and N400 amplitudes across phases than the amateurs. The results suggest that both the 1 h and long-term AVG experiences may be related to an increased difficulty of emotional perception. Furthermore, certain behavioral and ERP measures showed neither within- nor between-group differences, suggesting that the relationship between AVG experience and emotional perception skills still needs further research.

## 1. Introduction

Video gaming is becoming an increasingly important part of our entertainment experience. Population-based research suggests that 79.9% of 5- to 17-year-olds and 31.3% of adults spend some time on video gaming on a daily basis [1]. A report of the Entertainment Software Association shows that 63% of U.S. households surveyed have at least one frequent gamer (http://www.theesa.com/about-esa/esa-annual-report/). Video gaming offers a new approach to acquiring knowledge and to facilitating cognitive development, thus providing us with a new, important venue for understanding learning-related cognitive and neural plasticity. Using both behavioral and neuroscience methods, this study explores the relationship between action video gaming (AVG) experience and emotional perception performance.

A major genre of video gaming, AVG emphasizes physical and cognitive challenges, such as hand-eye coordination [2], stimulus detection and analysis [3], and decision-making under time pressure [4], just like traditional sports. Researchers have proposed that AVG experience induces plasticity of brain function and structure [5]. This proposition is supported by the findings that AVG experience is related to improvements in various cognitive functions (e.g., selective attention [6, 7], spatial distribution of visuospatial attention [8, 9], attentional capture [10], and behavioral performance in tasks of working memory and vision [11]) and the plasticity of brain structure and neutral network connectivity (e.g., increased gray matter volume of insula [12] and strengthened connection between sensorimotor and attentional networks [13]).

These studies have improved our knowledge of the influence of AVG experience on sensorimotor and attentional development—essential components of physical and cognitive development. However, human development consists of four domains: physical, cognitive, social, and emotional developments [14]. A holistic approach to human development encompasses all the four domains [14]. Then, an important question arises: How does AVG experience influence emotional perception development? An examination of this question is essential for any complete theories on the learning-related cognitive and brain plasticity.

This study examines the influence of AVG experience on the development of emotional perception skills. Essential to human experience [15], emotions guide us through complex social environment and help us adapt our behavior to optimize survival, health, and development [16]. Emotional information can be encoded visually and auditorily. For example, emotional information can be visually communicated through facial expressions [17], body postures [18], and written language [19]. Emotional information can also be encoded through the modulations of acoustic attributes (e.g., pitch, intensity, rate, stress, and pause) in speech, music, and even environmental sounds [20–22]. This study examines the decoding of emotional faces and emotional word meanings in both AVG experts and amateurs.

There are two competing positions on the effect of AVG experience on emotional skill development. One position suggests that video gaming experience may *hinder* emotional skill development [23]. For example, excessive video game use is related to low self-esteem [24], low self-efficacy [25], anxiety and aggression [26], and symptoms of depression and anxiety disorders [27], all contributing to socioemotional deficits. Video gaming experience is also related to a lack of real-life friends [28], stress and maladaptive coping [29], lower psychosocial well-being and loneliness [30], and psychosomatic problems [29, 31]. Video gaming experience may be inversely related to socioemotional skill development because research suggests that individuals with weak socioemotional skills may use video games to avoid real-life socioemotional problems [32] and that video gaming reduces the time allocated for real-life socioemotional interaction [33], thus decreasing the opportunity to develop adequate socioemotional skills. Furthermore, the exposure to the violent content of AVG may increase aggressive cognition and decrease empathy, thus hindering socioemotional skill development [34–36]. However, some of these findings are based on individuals with excessive video game use (e.g., [24–26]), who may have psychological issues that also contribute to socioemotional skill deficits [23]. For example, since the individuals with excessive video game use may have video gaming addiction, it is unclear whether their socioemotional skill deficits are due to video gaming experience per se or a general addiction effect.

However, a competing position suggests that video gaming experience may *improve* emotional skill development. Research suggests that playing puzzle and role-play video games can improve mood and increase positive emotion in the players (e.g., [37–39]). Furthermore, video games can be used to promote emotional intelligence in the 14-year-olds [40]. Video gaming may promote socioemotional skill development because it can help players relieve stress after real-life socioemotional problems (e.g., [38, 41]). Additionally, video gaming can improve emotional regulation strategies because it rewards overcoming frustration and anxiety [42]. Furthermore, team video gaming offers a social environment that can foster social bond, emotional communication, empathy, and prosocial behavior in players [43, 44]. Finally, a successful video gaming experience induces feelings of achievement in players—beneficial to emotional skill development [45]. All these socioemotional elements are relevant to AVG.

Thus, the effect of AVG experience on socioemotional skill development is still unclear. This study examines the relationship between action real-time strategy gaming (ARSG) experience and the perception of emotional faces and emotional word meanings. This study is aimed at determining whether emotional perception performance changes after a brief ARSG session within a participant and whether emotional perception performance differs between the ARSG experts and amateurs. ARSG is examined in this study because (i) as a new AVG subgenre that is highly popular, ARSG still remains understudied; (ii) ARSG includes “action” elements, allowing us to examine the effect of *action* video gaming on emotional skill development; and (iii) ARSG also includes “strategy” elements that offer players a socioemotional environment for socioemotional communication ([46]), thus allowing us to hypothesize that ARSG may enhance emotional skill development. Recently, research found that the “action” elements of ARSG may enhance the salience network (SN) and the central executive network (CEN)—two networks essential for attention and working memory [47], while the “strategy” elements of ARSG may enhance the functional integration between the CEN and the default mode network (DMN) [48]. However, these findings do not speak to emotional perception skills.

Using behavioral (i.e., response time, accuracy) and ERP measures, this study examined ARSG experts' and amateurs' performance in a Stroop task. This study used an established experimental procedure [49], referred to as an emotional face-word (EFW) Stroop task hereafter, in which participants see a face across which a word is projected—where the emotional meaning of the word and the facial expression is either congruent or incongruent—and are asked to decode the emotional meaning of the face *or* the word. A Stroop effect is the delay in response between the congruent and incongruent stimuli. An EFW Stroop task uses facial expressions and written texts because they are two primary channels of emotional communication. Facial expressions—realized through motions or positions of the muscles beneath the skin of the face—are a nonverbal means of emotional communication for humans [50], other mammals [51, 52], and species [53]. In addition, written language is a verbal means of emotional communication specific to humans [54].

This study used ERP measures because the high temporal resolution of ERP allows us to examine temporally sensitive indicators [55]. According to past findings [56–59], two ERP components (N400, N170) were analyzed in this study. The N400 was analyzed because (i) a clear N400 can be evoked by the incongruent color interference in a color-word Stroop task [56], (ii) the N400 may reflect the process of resolving the response conflict by suppressing the word response [57], and (iii) the N400 amplitude may increase with semantic and associative distance between a target word and its preceding context, suggesting that the N400 reflects the degree of conflict and thereby the processing difficulty [58]. Thus, the N400 is a sensitive measure of semantic processing in an EFW Stroop task. This study also analyzed the N170—an ERP component with a latency of about 170 ms poststimulus—because it is closely related to face perception and emotion processing, as the N170 may reflect late stages in the structural encoding of faces [59]. In addition, research has demonstrated that a clear N170 can be evoked in the incongruent condition in an EFW Stroop task [49]. Thus, the N170 can effectively indicate face perception in an EFW Stroop task.

In this study, both ARSG experts and amateurs completed a 1 h ARSG session. They were also administered an EFW Stroop task prior to (the pregaming phase) and after (the postgaming phase) the 1 h ARSG session. The influence of the 1 h ARSG session on emotional perception performance can be illuminated through cross-phase comparisons of the participants' pregaming and postgaming emotional perception performance. The influence of the long-term ARSG experience on emotional perception performance can be addressed through between-group comparisons of the participants' emotional perception performance. The interaction between the long-term and brief ARSG experience can be revealed by between-group comparisons of the amount of cross-phase change. If ARSG experience is related to a disadvantage of emotional perception, we predict that (i) cross-phase comparisons should show an increased response time, a decreased accuracy rate, and a more negative N400 and N170 in both groups of participants, all of which indicate an increased difficulty of emotional perception; (ii) compared to the amateurs, the experts should have a slower response time, a lower accuracy rate, and a more negative N400 and N170; and (iii) the experts should have a greater amount of cross-phase change than the amateurs. By contrast, if ARSG experience is related to improvements in emotional perception skills, an opposite pattern of results should emerge.

## 2. Method

### 2.1. Participants

Participants were recruited using the procedure established in previous research [60–62]. Prior to this study, a survey was given to a number of participants who were asked to report the following: (i) their video gaming experience (in years) and Expertise Ranking of League of Legends (LOL (the LOL, a video game that requires players to cooperate with teammates to destroy the opposing team's towers, is a typical ARSG game, which is also commonly referred to as the Multiplayer Online Battle Arena (MOBA) genre; the LOL consists of three game modes: Summoner's Rift (Ranked Matchmaking), Twisted Treeline, and Howling Abyss (Normal Matchmaking); players' gaming experience and expertise are indicated by their Ranked Matchmaking level which is calculated based on the Elo rating system—a method for calculating the relative skill levels of players in competitor-versus-competitor games; the experts and amateurs can be defined based on the Elo rating of their Ranked Matchmaking level, which is available from an online inventory (http://www.lol91.com/duanweibilv.html); the ELO rating system has multiple stages—Bronze, Silver, Gold, Platinum, Diamond, Master, and Challenger—across which the expertise level increases sequentially. Each stage has five phases ranging from V to I; in the Ranked Matchmaking mode, players who win (or lose) a game will gain (or lose) a certain amount of points depending on the champion's performance; wining each 100 points activates the promotion competition, the successful completion of which promotes the player to the next stage or level; the successful maintenance of points requires one to play LOL on a regular and frequent basis))—the ARSG program used in this study; (ii) the amount of time spent on LOL and other ARSGs in the most recent week; (iii) their LOL ID that was used to verify their self-reported LOL gaming experience and ranking information, since their LOL Expertise Ranking is provided by the LOL game; (iv) their experience (in years) of playing video games other than LOL, which was used to exclude multigenre gamers to ensure LOL was the primary game genre for all participants in this study; and (v) their video gaming addiction status, using a 7-item Game Addiction Scale. Only the individuals who were identified as either LOL experts or amateurs and who had no gaming addiction were invited to participate in this study. Unlike past research that recruited individuals who had no action video gaming experience [6, 63, 64], this study recruited amateurs who had some LOL experience to minimize the influence of novel experience on amateurs' performance. This is an established procedure used in past cross-sectional research where both experts and amateurs were administered a brief video gaming session [60].

The participants were 27 male college students (*M* = 20.5 ± 2.0 years) of the University of Electronic Science and Technology of China (UESTC), consisting of 13 LOL experts and 14 amateurs. Following the procedure established by previous research [60–62], the group membership was defined based on both time- and skill-based criteria. The experts had at least 2 years of LOL while the amateurs had less than 0.5 years of LOL experience. Furthermore, according to the Expertise Ranking information provided by the LOL software—ranging between rank 1 and rank 27, all the experts were above rank 17, and all the amateurs were below rank 8, thus further verifying their group membership. All participants spent around 7–21 hours on ARSG in the week when they participated in this study. In addition, all participants were right-handed as confirmed by the Edinburgh Handedness Questionnaire [65], reported having normal or corrected-to-normal vision, and had no history of neurological problems. This study complied with the ethical standards outlined by the Declaration of Helsinki and was approved by the UESTC Ethics Board and performed in accordance with the Code of Ethics of the World Medical Association ([66]). Informed consent was obtained from all participants prior to the experiment.

### 2.2. Stimuli and Procedure

An experiment consisted of three sequential phases: a pregaming Stroop test, a 1 h ARSG session, and a postgaming Stroop test. A monitor (Model Lenovo L2250PWD, 20 inches, height: 47.4 cm, wide^″^ 29.6 cm) was placed in the testing booth and used for stimulus presentation.

#### 2.2.1. Stimuli Preparation

**Figure 1:**
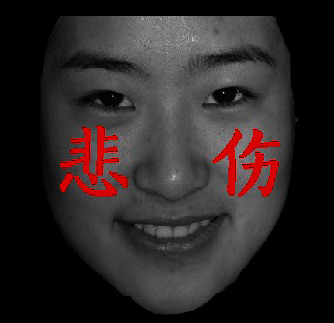
A sample stimulus in the emotional face-word Stroop task. The word on the face, which consists of two characters, means “sad”.

The stimuli were presented using the established EFW Stroop procedure [49]. The stimuli were selected from an existent face database designed for an EFW Stroop task [49]. Based on the valence of emotion [67], a face was either positive (e.g., happiness), or negative (e.g., sad), or neutral (e.g., calmness). A two-character Chinese word in prominent red color was projected across each face, one character on each side of the face (Figure 1) [49]. The word was either an emotional adjective (e.g., “愉快” [“yu-kuai” meaning happy], “悲伤” [“bei-shang” meaning sad], or “镇定” [“zhen-ding” meaning calm]) or a noun without an explicit emotional connotation (e.g., “苹果” [“ping-guo” meaning apple]). There were three types of trials: (i) *congruent trials* where the valence of the face and the word meaning was both positive, or both negative, or both neutral; (ii) *incongruent trials* where the valence of the face and the word meaning was inconsistent (e.g., a happy face paired with an adjective of “悲伤” [sad] or “镇定” [calm]); and (iii) *unrelated trials* where a face was paired with a noun (e.g., “苹果” [apple]). The size of the face was about 5 cm × 7 cm, and the size of the character was about 1.5 cm × 1.5 cm, when presented on the computer screen.

#### 2.2.2. Emotional Face-Word (EFW) Stroop Task

Each EFW task had four blocks (two face blocks and two word blocks), each containing 96 trials. In a face block, the participants were asked to read the word loudly and respond as quickly and accurately as possible by pressing one of the three buttons on the computer keyboard corresponding to “positive,” “negative,” and “neutral” for the effect expressed on the face. The participants responded to the facial expression while the valence of the word meaning was either in conflict with, or consistent with, or irrelevant to that of the facial expression. Thus, a face block was in essence a face perception task. Therefore, the N170—which is closely related to face perception [49]—was used as the ERP measure in the face blocks.

In a word block, the participants were asked to first orally describe the face using an adjective they thought about and then judge the word's emotional connotation using the keyboard as quickly and accurately as possible. The participants responded to the word meaning while the valence of the facial expression was either in conflict with, or consistent with, or irrelevant to that of the word meaning. Thus, a word block was in essence a semantic processing task. Therefore, the N400—which is closely related to semantic processing [68, 69]—was used as the ERP measure in the word blocks.

The face blocks and the word blocks differed in the response target in the Stroop task (face vs. word) and the source of the potential distraction (word vs. face). Thus, we used different ERP measures in the two types of blocks. Nevertheless, both types of blocks assessed emotional perception with potential conflict information.

#### 2.2.3. Procedure

The participants were tested individually in a sound attenuated, dim lit, and electrically shielded testing booth. To familiarize them with the Stroop task, an experiment started with a familiarization phase containing seven word trials and seven face trials. Each trial started with the presentation of a fixation square at the center of the display for 1 s. Then, the test stimulus appeared at the center of this display. The presentation of the test stimulus, which could maintain for a maximal duration of 8 s, terminated once the participant made a response. Then, the next trial started after a random response-stimulus interval (RSI), ranging from 0.85 s to 1.25 s. The familiarization phase ended with a 10 m break, during which the participants were asked to rest with their eyes closed.

*(1) Pregaming Stroop Task*. The experiment then proceeded to the pregaming EFW Stroop task that contained two face blocks and two word blocks. The presentation order of the trials within a block was randomized within a participant and counterbalanced across participants. Two presentation orders of blocks were used (order 1: a face block, a word block, a word block, a face block; order 2: a word block, a face block, a face block, a word block). There was a 10 s break after each block.

*(2) A 1 h ARSG Session*. The participants then completed a 1 h LOL session, using the Normal 5v5 Matchmaking—a LOL game model referred to as Howling Abyss (version: 8.5)—where two teams, each consisting of five members, competed with each other. Each player controlled a character to destroy the opponent team's tower by working with his four teammates. Since the participants were tested individually, the feature “Random Pick” was activated to randomly team the participant with four online players who were also playing LOL at that given time regardless of their physical location, competing against the opponent team who was also randomly assigned by LOL. The feature “Random Pick” ensured that all 10 players in a 1 h LOL session had a similar level of experience, expertise, and ranking.

*(3) Postgaming Stroop Task*. Finally, the participants completed another EFW Stroop task. The procedure of the postgaming EFW Stroop task was identical to that of the pregaming Stroop task, except that the presentation orders of the four blocks and the test trials within each block differed across phases.

### 2.3. Data Recording and Preprocessing

The EEG data was collected using an electrode cap (Brain Products GmbH) of 32 Ag-AgCl electrodes following the 10-20 system [70]. Contact impedance was kept below 5k*Ω*. The EEG signals were digitized with a sampling rate of 1000 Hz and filtered through 50 Hz notch filter. To control eye movement artifacts, vertical electrooculogram (EOG) was recorded above the right eye. All recording was completed through Brain Vision Record version 2.0.1 (Brain products GmbH), and off-line EEG analysis was performed using Brain Vision Analysis version 2.0.1 (Brain products GmbH).

EEG signals were rereferenced to average reference and digitally band-pass filtered between 0.1 and 30 Hz using FIR filter. Then, raw EEG signals were segmented into two parts according to the first and the second Stroop tasks. To remove EOG artifacts, the segmented data was corrected through ocular correction, excluding all signal of which amplitude exceeded 90 *μ*V in any channel from additional analysis. After raw data inspection, all epochs under “incongruent” condition were extracted as 1000 ms (200 ms prestimulus and 800 ms poststimulus) for further analysis. For each epoch, baseline correction for the period 200 ms before the appearance of stimulus was applied. Averaging epochs of face blocks and word blocks, respectively, in the first and the second tasks, we can obtain four ERPs (see details in 2.5) of each participant as the research objects. In every group, participants' ERPs were averaged again for contrasting the two groups.

### 2.4. Behavioral and ERP Analysis

In this study, data analysis and reporting were focused on the incongruent condition because it is where the Stroop interference effect occurred. In total, 1248 trials (13 participants × 96 trials) were averaged for the pre- and postgaming EFW Stroop tasks, respectively, within the experts, and 1344 trials (14 participants x 96 trials) were averaged the pre- and postgame EFW Stroop tasks, respectively, within the amateurs. This study used two behavioral dependent variables—response time (i.e., the delay between stimuli onset and button pressing) and accuracy rate—which were recorded by E-prime Data analysis. The ERP analysis was focused on the N170 (for the face blocks [71]) and the N400 (for the word blocks [72, 73]). Based on the time of stimulus appearance as time-locked point, the average amplitude was calculated according to the time window and recording electrodes as shown in Table 1.

### 2.5. Statistical Analysis

A 2 (Group : experts, amateurs) × 2 (Phase : pregaming, postgaming) repeated measures ANOVA was conducted. If significant main effects or a Group × Phase interaction emerged, post hoc analyses were conducted through paired sample *t*-tests on cross-phase differences within each group and/or independent samples *t*-tests on between-group differences. Adjusted *p* values were used in multiple comparisons throughout this study. This study used cross-phase comparisons to explore the effect of the 1 h ARSG session on emotional perception performance, and between-group comparisons to examine the effect of the long-term ARSG experience on emotional perception performance.

## 3. Results

### 3.1. Behavior Results

#### 3.1.1. The Face Blocks

A 2 (Group) × 2 (Phase) repeated measures ANOVA on response time found that the main effect of Group was not significant (*F*(1, 25) = 0.12, *p* = 0.74), suggesting that response time did not differ between groups. Furthermore, the main effect of Phase was significant (*F*(1, 25) = 11.67, *p* = 0.002), but the Group × Phase interaction (*F*(1, 25) = 0.001, *p* = 0.85) was not significant, suggesting that responses time differed across phases and that the same pattern of results emerged in the two groups. Then, paired sample *t*-tests showed that response time decreased across phases in both groups (Table 2). In addition, a 2 (Group) × 2 (Phase) repeated measures ANOVA on accuracy rate showed that neither the main effects of Group (*F*(1, 25) = 1.94, *p* = 0.18) and Phase (*F*(1, 25) = 1.93, *p* = 0.18) nor the Group × Phase interaction (*F*(1, 25) = 0.28, *p* = 0.60) approached significance, suggesting that accuracy rate did not differ between groups or across phases (the experts: *M*pre = 92.17% ± 4.3, *M*post = 91.24% ± 6.3; the amateurs: *M*pre = 89.45% ± 5.9; *M*post = 87.72% ± 5.4).

#### 3.1.2. The Word Blocks

A 2 (Group) × 2 (Phase) repeated measures ANOVA on response time found that the main effect of Group (*F*(1, 25) = 0.12, *p* = 0.73) was not significant, suggesting that response time did not differ between groups. Furthermore, the main effect of Phase was significant (*F*(1, 25) = 56.44, *p* < 0.001), but the Group × Phase interaction (*F*(1, 25) = 0.007, *p* = 0.94) was not significant, suggesting that response times differ across phases and that the same pattern of results emerged in the two groups. Then, paired sample *t*-tests found that response time decreased across phases in both groups (Table 3). In addition, a 2 (Group) × 2 (Phase) repeated measures ANOVA on accuracy rate found that neither the main effects of Group (*F*(1, 25) = 2.94, *p* = 0.10) and Phase (*F*(1, 25) = 0.81, *p* = 0.38) nor the Group × Phase interaction (*F*(1, 25) = 0.26, *p* = 0.62) approached significance, suggesting that accuracy rate did not differ between groups or across phases within a group (the experts: *M*pre = 96.76% ± 3.4, *M*post = 96.43% ± 3.3; the amateurs: *M*pre = 94.83% ± 2.4; *M*post = 93.63% ± 6.5) (Table 3).

Thus, the behavioral data showed that neither response time nor accuracy rate differed between groups. In addition, both the experts' and amateurs' response time decreased after the 1 h gaming session.

### 3.2. N170 (the Face Blocks)

**Figure 2:**
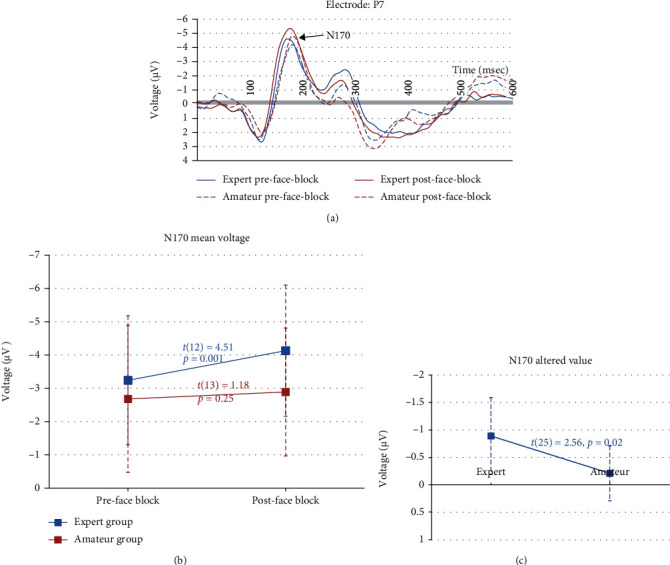
(a) The N170 in the face block: solid blue lines (experts in the pregaming phase), red solid lines (experts in the postgaming phase), blue dashed lines (amateurs in the pregaming phase), and red dashed lines (amateurs in the postgaming phase). (b) The paired sample *t-*test results that the N170 amplitude increased across phases in the experts but not in the amateurs. (c) The independent sample *t-*test results that the altered values of the N170 amplitude were greater in the experts than in the amateurs.

The N170 was recorded by the P7 electrode.  Figure 2 shows the ERP contrast between the pre- and postgaming Stroop tasks on the incongruent face trials in the experts and amateurs.  Table 4 shows the descriptive and deductive results. A 2 (Group) × 2 (Phase) repeated measures ANOVA on the N170 amplitude showed that the main effect of Group (*F*(1, 25) = 1.38, *p* = 0.25) was not significant, but the main effect of Phase (*F*(1, 25) = 17.33, *p* < 0.001) and the Group × Phase interaction (*F*(1, 25) = 6.67, *p* = 0.02) were significant. To decompose this interaction, paired sample *t*-tests compared the N170 amplitude across phases within each group. Results showed that the N170 amplitude increased across phases in the experts but not in the amateurs (Table 4). Then, separate independent samples *t*-tests found that the N170 amplitude did not significantly differ between groups in either the pre- or postgaming phase. However, an independent samples *t*-test on the altered value of the N170 amplitude (the postgaming phase data minus the pregaming phase data) showed that the experts had a greater altered value than the amateurs.

### 3.3. N400 (the Word Blocks)

**Figure 3:**
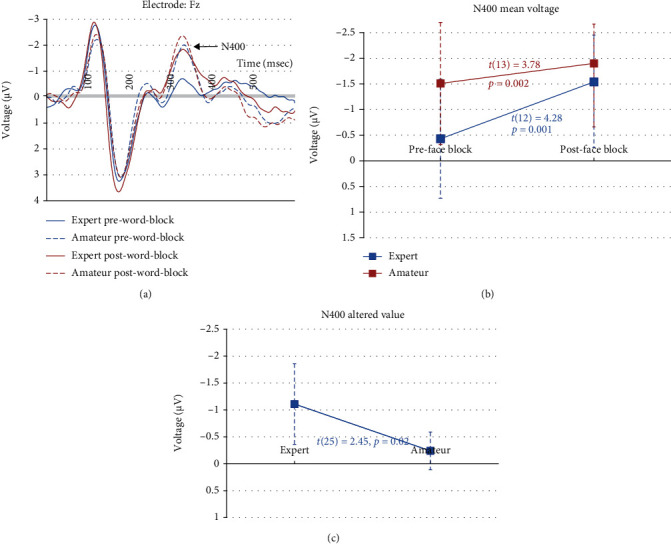
(a) The N400 in the word block: solid blue lines (experts in the pregaming phase), red solid lines (experts in the postgaming phase), blue dashed lines (amateurs in the pregaming phase), and red dashed lines (amateurs in the postgaming phase). (b) The paired sample *t-*test results that the N400 amplitude increased across phases in both groups of participants. (c) The independent sample *t-*test results that the altered values of the N400 amplitude were greater in the experts than in the amateurs.

The N400 was recorded by the Fz electrode [73].  Figure 3 shows ERP contrast between the pre- and postgaming Stroop tasks on the incongruent word trials in the experts and amateurs.  Table 5 shows the descriptive and deductive results. A 2 (Group) × 2 (Phase) repeated measures ANOVA on the N400 amplitude showed that the main effect of Group (*F*(1, 25) = 2.62, *p* = 0.12) was not significant, but the main effect of Phase (*F*(1, 25) = 30.74, *p* < 0.001) and the Group × Phase interaction (*F*(1, 25) = 6.99, *p* = 0.01) were significant. To decompose this interaction, paired sample *t*-tests compared the N400 amplitude across phases within each group. Results showed that the N400 amplitude increased across phases in both groups (Table 5). Then, separate independent samples *t*-tests found that the N400 amplitude was greater in the amateurs than in the experts in the pregaming phase, but it did not differ between groups in the postgaming phase. In addition, an independent *t* samples test on the altered value of the N400 amplitude (the postgaming task data minus the pregaming task data) showed that the experts had a greater altered value than the amateurs (past research also used N2 and MFN as the index of the proactive cognitive control in a Stroop task; however, the current study found no significant between- or within-group differences for either N2 (recorded by Fz) or MFN (recorded by Cz)).

## 4. Discussion

This study examined the relationship between ARSG experience and emotional perception performance. The ARSG experts and amateurs were administered an EFW Stroop task prior to (the pregaming phase) and after (the postgaming phase) a 1 h ARSG session. The influence of the 1 h ARSG session on emotional perception performance was examined through cross-phase comparisons within each group, while the influence of the long-term ARSG experience on emotional perception performance was explored through between-group comparisons.

### 4.1. Was the 1 h ARSG Experience Related to Emotional Perception Performance?

Results showed that the N170 amplitude increased across phases in the experts but not in the amateurs. In an EFW Stroop task, the N170 amplitude may reflect recognition difficulty and cognitive resource consumption for processing emotional faces [49]. Indeed, an increased N170 amplitude is often observed in the incongruent condition in a Stroop task [49]. The face-language conflict may complicate emotional face perception, leading to an increased difficulty of emotional face perception that requires a greater cognitive resource consumption [49]. Thus, the current findings suggest that after the 1 h ARSG session, an increased difficulty of emotional processing of facial expressions may be observable in the experts but not in the amateurs. Perhaps, compared to the amateurs, the experts processed more gaming information in the 1 h ARSG session, which might have biased their brain toward a greater allocation of cognitive resources for gaming information perception. This bias might have maintained in the postgaming phase, thereby deprioritizing the experts' perception of nongaming information (e.g., emotion) in the postgaming Stroop task.

Furthermore, a more negative N400 was observed across phases in both groups of participants, revealing an increased difficulty of emotional perception of word meaning after the 1 h ARSG session. The N400 is a classic ERP component in response to semantic anomaly [74]. The N400 can also be elicited by word-picture incongruency [68] and inaccurate arithmetic results [69]. Furthermore, EFW Stroop research found a stronger N400 in the incongruent condition than in the congruent condition [57], suggesting that the N400 may reflect the degree of incongruency in the Stroop paradigm. Perhaps, video gaming prioritized the processing of gaming information, which decreased the allocation of cognitive resources for emotional perception in both the experts and amateurs. This pattern of cognitive resource allocation might have maintained in the postgaming phase, leading to an increased difficulty of emotional perception and thereby a more negative N400 in both groups of participants.

Thus, the current findings confirmed the prediction that the 1 h ARSG session may be related to an increased difficulty of emotional perception of word meaning in both groups of participants according to the N400 data. Furthermore, the 1 h ARSG session may be related to an increased difficulty of emotional perception of facial expressions in the experts according to the N170 data, suggesting that the effect of a brief ARSG session on emotional perception performance may interact with one's long-term gaming experience. However, it is unclear whether the increased difficulty of emotional perception was due to the 1 h action video gaming session per se or a general screen media experience unspecific to video gaming. Future research should evaluate this issue.

### 4.2. Was the Long-Term ARSG Experience Related to Emotional Perception Performance?

This study used between-group comparisons to evaluate the effect of the long-term ARSG experience on emotional perception performance. Results showed that the altered values of the N400 and N170 amplitude across phases (postgaming Stroop task minus pregaming Stroop Task) were greater in the experts than in the amateurs, suggesting that the experts had a greater increase in the difficulty of emotional perception than the amateurs after the 1 h ARSG session.

However, there is also evidence against the claim that ARSG experience is related to an increased difficulty of emotional perception. First, neither response time nor accuracy rate differed between groups. Nevertheless, these behavioral measures may not be sensitive enough to reveal the potential between-group differences in emotional perception performance. Second, the N170 amplitude did not differ between groups in either the pregaming phase or the postgaming phase. In addition, the N400 amplitude did not differ between groups in the postgaming phase. These results do not support the prediction that long-term AVG experience is related to emotional perception performance. Third, we even found a more negative N400 in the amateurs than in the experts in the pregaming Stroop task, suggesting that the amateurs might have a greater difficulty of emotional perception of word meaning than the experts in the pregaming phase, which is inconsistent with the altered value data showing that long-term AVG experience is related to an increased difficulty of emotional perception. It should be noted that although past research suggests that compared with amateurs, AVG experts have faster processing and responses in space detection [75], task switching [76], or could better detect changes to objects stored in visual short-term memory [76], these findings are not about emotional perception. Nevertheless, these findings suggest that the relationship between AVG experience and emotional perception skills still requires further research.

## 5. Conclusions

Using behavioral and ERP measures, this study explored the relationship between AVG experience and the ability to decode emotional meaning of facial expressions and emotional words. This study revealed evidence that both the brief and long-term AVG experiences may be related to an increased difficulty of emotional perception. Nevertheless, the relationship between AVG experience and emotional perception skills still needs further research.

## Figures and Tables

**Table 1:** Components, electrodes, and time window used in ERP analyses.

Component	N170	N400
Electrode	P7	Fz
Time window (ms poststimulus)	150-200	300-360

**Table 2:** Descriptive and deductive results of response time in the face blocks.

	Experts	Amateurs
Pretask response time (ms)	*M* = 1380.64, SD = 230.5	*M* = 1381.72, SD = 209.2
Posttask response time (ms)	*M* = 1283.82, SD = 215.2	*M* = 1301.81, SD = 168.4
Paired sample *t*-test between the pre- and postgaming Stroop tasks within each group	*t*(12) = 2.15, *p* = 0.049 (marginal significance, as a significance cut-off level of 0.025, was used)	*t*(13) = 2.79, *p* = 0.02

**Table 3:** Descriptive and deductive results of response time in the word blocks.

	Experts	Amateurs
Pregaming response time (ms)	*M* = 2023.57, SD = 362.6	*M* = 2064.96, SD = 281.1
Postgaming response time (ms)	*M* = 1787.31, SD = 310.9	*M* = 1823.57, SD = 293.8
Paired sample *t*-test between the pre- and postgaming Stroop tasks within each group	*t*(12) = 6.92, *p* < 0.001	*t*(13) = 4.41, *p* < 0.001

**Table 4:** Descriptive and deductive results of N170 in the face blocks.

	Experts	Amateurs
Pregaming	*M* = −3.24, SD = 1.94	*M* = −2.68, SD = 1.97
Postgaming	*M* = −4.13, SD = 2.21	*M* = −2.89, SD = 1.92
Paired sample *t*-tests between pre- and postgaming Stroop tasks within each group	*t*(12) = 4.51, *p* = 0.001	*t*(13) = 1.18, *p* = 0.25
Altered value (postgaming Stroop task minus pregaming Stroop task)	-0.89	-0.21
Independent samples *t*-test between experts and amateurs	Pregaming: *t*(25) = .74, *p* = 0.46	
	Postgaming: *t*(25) = 1.53, *p* = 0.14	
	Altered value: *t*(25) = 2.56, *p* = 0.02	

**Table 5:** Descriptive and deductive results of N400 in the word blocks.

	Experts	Amateurs
Pregaming Stroop task	*M* = −0.43, *SD* = 1.16	*M* = −1.51, *SD* = 1.28
Postgaming Stroop task	*M* = −1.54, *SD* = 1.19	*M* = −1.90, *SD* = 1.17
Paired sample *t*-test between pre- and postgaming Stroop tasks	*t*(12) = 4.28, *p* = 0.001	*t*(13) = 3.78, *p* = 0.002
Altered value (postgaming Stroop task minus pregaming Stroop task)	-1.11	-0.24
Independent samples *t*-test between experts and amateurs	Pregaming: *t*(25) = 2.29, *p* = 0.03	
	Postgaming: *t*(25) = 0.79, *p* = 0.43	
	Altered value: *t*(25) = 2.45, *p* = 0.02	

## Data Availability

Data available are on request.
